# Phylogenomic reconstruction of lactic acid bacteria: an update

**DOI:** 10.1186/1471-2148-11-1

**Published:** 2011-01-01

**Authors:** Zhi-Gang Zhang, Zhi-Qiang Ye, Li Yu, Peng Shi

**Affiliations:** 1 State Key Laboratory of Genetic Resources and Evolution, Laboratory of Evolutionary and Functional Genomics, Kunming Institute of Zoology, Chinese Academy of Sciences, Kunming 650223, PR China; 2 Laboratory for Conservation and Utilization of Bio-resource & Key Laboratory for Microbial Resources, Ministry of Education, Yunnan University, PR China; 3 Graduate School of the Chinese Academy of Sciences, Beijing, PR China

## Abstract

**Background:**

Lactic acid bacteria (LAB) are important in the food industry for the production of fermented food products and in human health as commensals in the gut. However, the phylogenetic relationships among LAB species remain under intensive debate owing to disagreements among different data sets.

**Results:**

We performed a phylogenetic analysis of LAB species based on 232 genes from 28 LAB genome sequences. Regardless of the tree-building methods used, combined analyses yielded an identical, well-resolved tree topology with strong supports for all nodes. The LAB species examined were divided into two groups. Group 1 included families Enterococcaceae and Streptococcaceae. Group 2 included families Lactobacillaceae and Leuconostocaceae. Within Group 2, the LAB species were divided into two clades. One clade comprised of the acidophilus complex of genus *Lactobacillus *and two other species, *Lb. sakei *and *Lb. casei*. In the acidophilus complex, *Lb. delbrueckii *separated first, while *Lb. acidophilus*/*Lb. helveticus *and *Lb. gasseri*/*Lb. johnsonii *were clustered into a sister group. The other clade within Group 2 consisted of the salivarius subgroup, including five species, *Lb. salivarius*, *Lb. plantarum*, *Lb. brevis*, *Lb. reuteri*, *Lb. fermentum*, and the genera *Pediococcus, Oenococcus*, and *Leuconostoc*. In this clade, *Lb. salivarius *was positioned most basally, followed by two clusters, one corresponding to *Lb. plantarum*/*Lb. brevis *pair and *Pediococcus*, and the other including *Oenococcus*/*Leuconostoc *pair and *Lb. reuteri*/*Lb. fermentum *pair. In addition, phylogenetic utility of the 232 genes was analyzed to identify those that may be more useful than others. The genes identified as useful were related to translation and ribosomal structure and biogenesis (TRSB), and a three-gene set comprising genes encoding ultra-violet resistance protein B (*uvrB*), DNA polymerase III (*polC*) and penicillin binding protein 2B (*pbpB*).

**Conclusions:**

Our phylogenomic analyses provide important insights into the evolution and diversification of LAB species, and also revealed the phylogenetic utility of several genes. We infer that the occurrence of multiple, independent adaptation events in LAB species, have resulted in their occupation of various habitats. Further analyses of more genes from additional, representative LAB species are needed to reveal the molecular mechanisms underlying adaptation of LAB species to various environmental niches.

## Background

Lactic acid bacteria (LAB) are Gram-positive bacteria that have been widely used as starter or nonstarter cultures in the plant, meat, and dairy fermentation, and also as probiotic bacteria in human gastrointestinal tract contributing to pathogen inhibition and immunomodulation. At present, nearly 400 LAB species have been recognized [1]. They are generally classified into four families and seven genera, as follows: family Lactobacillaceae (genera *Lactobacillus *and *Pediococcus*), family Leuconostocaceae (genera *Oenococcus *and *Leuconostoc*), family Enterococcaceae (genus *Enterococcus*) and family Streptococcaceae (genera *Lactococcus *and *Streptococcus*) [2-4]. Phylogenetic relationships among the LAB species have been hotly disputed. One of the foremost debates in LAB phylogeny concerns the species in the genera *Lactobacillus, Pediococcus, Oenococcus*, and *Leuconostoc*, which belong to family Lactobacillaceae and Leuconostocaceae, due to the severe disagreements arising from analyses of different data sets [2-11]. In the genus *Lactobacillus*, there are uncertainties about the interspecies affinities within the acidophilus complex [8] that consists of five species *Lb. gasseri, Lb. johnsonii, Lb. acidophilus, Lb. helveticus *and *Lb. delbrueckii*. In particular, the divergence between *Lb. gasseri*/*Lb. johnsonii*, *Lb. acidophilus*/*Lb. helveticus *and *Lb. delbrueckii *remains unresolved. Based on the analyses of a 16 S rRNA gene and a few nuclear genes [3,5,7,10,12] and that of 32 ribosomal proteins [9], *Lb. delbrueckii *was found to be more closely associated with *Lb. acidophilus*/*Lb. helveticus *than with *Lb. gasseri*/*Lb. johnsonii*. However, a recent study using 141 core proteins from 17 LAB species suggested that *Lb. delbrueckii *diverged earliest within the acidophilus complex, while *Lb. acidophilus*/*Lb. helveticus *and *Lb. gasseri*/*Lb. johnsonii *clustered into a sister group [8].

Although the paraphyly of *Lactobacillus species *is well-established, a general consensus for the placement of the *Lactobacillus *species, e.g., *Lb. salivarius*, *Lb. plantarum*, *Lb. brevis*, *Lb. reuteri*, *Lb. sakei*, and *Lb. casei*, and their relationship to the genera *Pediococcus, Oenococcus*, and *Leuconostoc *has not yet emerged in the 'salivarius' subgroup. For example, in the analysis of four subunits of RNA polymerase, the clade uniting *Lb. sakei *and *Lb. casei *is placed at the most basal position, followed by *Lb. salivarius. Pediococcus *is sister to the clade containing *Lb. plantarum *and *Lb. brevis*, while *Oenococcus*/*Leuconostoc *clusters with *Lb. reuteri *[7]. In contrast, an analysis of 141 core proteins suggested that the *Lb. sakei*/*Lb. casei *clade is more related to acidophilus complex, while the other *Lactobacillus *species and *Pediococcus, Oenococcus*, as well as *Leuconostoc *group together, in which *Oenococcus*/*Leuconostoc *diverged earliest, followed by *Lb. salivarius*, *Pediococcus*, *Lb. reuteri*, and lastly the species most recently diverged, *Lb. plantarum *and *Lb. brevis *[8].

These findings highlight the need to gather and analyze larger sequence data sets in order to unravel the phylogenetic relationships among LAB species and clarify specifically those within genera *Lactobacillus, Pediococcus, Oenococcus*, and *Leuconostoc*. The increasing availability of LAB genome sequence data provides a good opportunity to understand the evolutionary history of LAB species. In the present study, we studied LAB phylogeny by gathering and analyzing 232 orthologous genes from 28 LAB genome sequences representing all genera from four families. Our objectives were to provide new insights into the relationships of LAB species and to examine the utility of such an analysis in the context of LAB phylogeny, and develop new potential genetic markers for study of LAB systematics. This study not only contributes to clarifying the currently obscure LAB species relationship, but also lays a foundation for further studies on adaptive evolution of LAB species in different environmental niches.

## Results and Discussions

### Identification of orthologous genes

The use of accurate and reliable methods for the identification of orthologous genes is essential for phylogenetic reconstruction based on analyses of large data sets, especially for those using whole genome sequences [13]. In the present study, the strategy of developing potential orthologous gene sets for LAB phylogenomic studies was different from those used in previous LAB analyses. First, in previous studies of LAB phylogeny, less stringent clusters of orthologous groups (COGs) [6] and reciprocal best hits [8] methods were applied to identify putative orthologs. Here, we applied both Inparanoid [14] and MultiParanoid [15] programs to serve this purpose. Inparanoid [14] exploits a BLAST-based strategy to identify orthologs as reciprocal best hits between two species, and applies additional rules to accommodate in-paralogs that arise from recent duplication events after speciation. Compared with other methods, including COGs [16] and OrthoMCL [17], Inparanoid's superiority lies in the ability to distinguish orthologs from in-paralogs and out-paralogs (those that arose via ancient duplication event before speciation) [17-21]. MultiParanoid software [15] performs clustering of orthologs and in-paralogs that are shared by more than two species. By using the conservative searching algorithms, we obtained a total of 310 one-to-one protein coding orthologs (Additional file 1 Table S1).

To make our dataset more conservative, we further excluded potentially problematic orthologs such as those with short sequence lengths and those involved in horizontal gene transfer (HGT). These criteria have not yet been used in previous LAB studies. In the end, a total of 232 orthologous genes, including 225 genes that have clear functional definition and 7 genes that have been annotated as hypothetical proteins (Additional file 2 Table S2), were used to reconstruct LAB phylogeny in this study. This dataset of 232 genes included those encoding 135 out of the 141 core proteins of the Claesson's study [8] that were identified by phylogenomic analyses of 17 LAB species genomes. Noticeably, 6 core proteins included in Claesson's study [8] were discovered as in-paralogs here and hence excluded from further analyses. This suggests that our dataset is more conservative and reliable than those from previous studies aimed at inferring LAB phylogeny.

### Reconstruction of LAB phylogenomic tree

**Figure 1:**
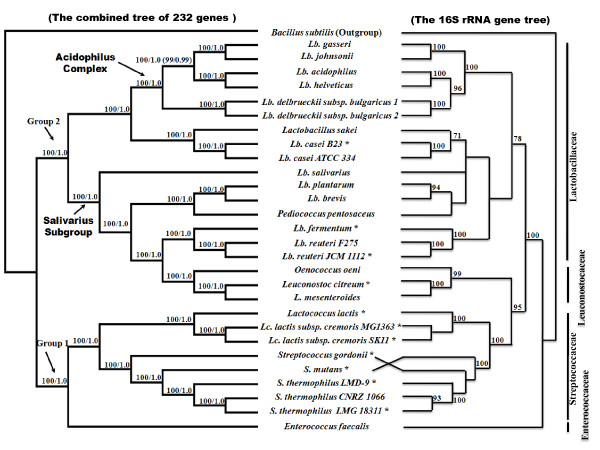
**Partitioned Bayesian/ML tree topology inferred from the selected 232 genes and the 16 S rRNA gene tree of 29 species**. For the concatenated tree of 232 genes, ML bootstrap supports and Bayesian posterior probabilities are shown above the branches. The stars imply newly added species in this study compared with that of Claesson et al. [8]. *Lb. delbrueckii subsp. bulgaricus *1 refers to *Lb. delbrueckii subsp. bulgaricus *ATCC BAA-365; *Lb. delbrueckii subsp. bulgaricus *2 refers to *Lb. delbrueckii subsp. bulgaricus *ATCC 11842; NJ analysis under 1000 bootstrap runs of 16 S rRNA genes from the study by Ventura et al [12] and Kawamura et al 's study [22]. ML bootstrap supports higher than 50 are shown above the branches.

Based on the concatenated amino acid alignment of 232 genes, phylogenetic analyses using two gap selection criteria (see Methods) and two tree-building methods, partitioned maximum likelihood (ML) and Bayesian analyses, yielded an identical, well-resolved tree topology with strong supports for all nodes (BS > 99% and PP > 0.99) (Figure 1), suggesting that the accuracy of our phylogenetic inference is independent of tree-building methods. As revealed in Figure 1 the monophyly for families Leuconostocaceae, Enterococcaceae and Streptococcaceae were strongly supported. For Lactobacillaceae, some species were more closely related to Leuconostocaceae than the other Lactobacillaceae species, supporting the paraphyly for family Lactobacillaceae, providing a possibility that Leuconostocaceae and Lactobacillaceae can be combined into a family.

The LAB species were divided into two groups. Group 1 included Enterococcaceae and Streptococcaceae. Group 2 included Lactobacillaceae and Leuconostocaceae. Within Group 1, the monophyly of the genera *Enterococcus*, *Lactococcus *and *Streptococcus *were strongly supported. In *Streptococcus*, *S. mutans *and *S. thermophilus *were grouped together, and *S. gordonii *was their sister taxon. The relationships within Group 1 observed here were congruent with two other studies [5,10], but disagreed with the 16 S rRNA gene tree [22] (Figure 1). Within Group 2, LAB species were divided into two clades. One clade composed of acidophilus complex of genus *Lactobacillus *and two other *Lactobacillus *species, *Lb. sakei *and *Lb. casei*. This result is in contradiction with the RNA polymerase-based study of Liu [7] that suggested that *Lb. sakei *and *Lb. casei *are more closely related to other *Lactobacillus *species and the genera *Pediococcus*, *Oenococcus *as well as *Leuconostoc*. However, our results are in agreement with the RNA polymerase trees [5,10], ribosomal-protein tree [9] and the 141-core proteins tree [8]. Of the five recognized *Lactobacillus *species in the acidophilus complex, our results strongly support the notion that *Lb. delbrueckii *separated first, while *Lb. acidophilus*/*Lb. helveticus *and *Lb. gasseri*/*Lb. johnsonii *clustered into a sister group. This finding is in accordance with the result derived from the 141-core proteins analyses [8], but disagrees with those derived the single 16 S rRNA gene [3,8,12] and the nuclear gene analyses [5,7,10,23] as well as that of 32 ribosomal proteins [9], in which *Lb. delbrueckii *was seen to be more closely associated with *Lb. acidophilus*/*Lb. helveticus *than *Lb. gasseri*/*Lb. johnsonii*. Five *Lactobacillus *species, including *Lb. salivarius*, *Lb. plantarum*, *Lb. brevis*, *Lb. reuteri*, *Lb. fermentum*, and the genera *Pediococcus, Oenococcus*, and *Leuconostoc *constitute the other clade, the 'salivarius' subgroup within Group 2. In this clade, *Lb. salivarius *was positioned most basally, followed by two distinct clusters, one corresponding to *Lb. plantarum*/*Lb. brevis *group and *Pediococcus*, and the other including *Oenococcus*/*Leuconostoc *group and *Lb. reuteri*/*Lb. fermentum *group. The basal position of *Lb. salivarius *in this clade is consistent with the RNA polymerase tree inferred by Makarova and Koonin [5] as well as by Liu [7], but not with the 16 S rRNA gene tree [12] and studies by Claesson [8] and Cai [10] that indicated that *Oenococcus*/*Leuconostoc *group diverged first. In addition, the grouping of *Lb. plantarum*/*Lb. brevis *and *Pediococcus *observed here is supported in most current studies, but is in contradiction with the recent proposal of the connecting of *Lb. plantarum*/*Lb. brevis *and *Lb. reuteri*. In the present study, the close relatedness of *Oenococcus*/*Leuconostoc *group and *Lb. reuteri*/*Lb. fermentum *is in agreement with RNA polymerase tree inferred by Liu et al. [7]. The possible placement of *Oenococcus*/*Leuconostoc *group as the first diverging taxa [8,10] or as the diverging taxa subsequent to *Lb. salivarius *[5] was not supported here.

Taken together, our study provides new insights into the evolutionary relationships of these LAB species, and helps to resolve the current controversial issues in LAB phylogeny. Depending on the gene segments or genomes and the tree-building methods used, different phylogenetic hypotheses can be obtained. Interestingly, our study demonstrated that different evolutionary rates among sites may also affect LAB phylogenetic reconstruction. When we repeated the phylogenetic analyses by setting a fixed alpha value of gamma distribution in the optimal amino acid substitution model, the species relationships within acidophilus complex, i.e., that among *Lb. gasseri*/*Lb. johnsonii*, *Lb. acidophilus*/*Lb. helveticus *and *Lb. delbrueckii*, became unstable and were poorly supported in partitioned ML and Bayesian analyses (data not shown). Therefore, our study revealed that different evolutionary rate among sites is also an important factor in tracing the evolutionary history of LAB species.

Besides the contribution of phylogenetic resolution, our results revealed the presence of independent adaptation to four types of habitat niches in LAB species (Figure 1), involving human gastrointestinal tract, human oral flora, dairy fermentation and other fermentations of beer, wine, plants, or meat (Table 1). For example, within acidophilus complex, *Lb. acidophilus *that is isolated from human gastrointestinal tract and *Lb. helveticus *that is widely applied to dairy fermentation are more closely related to each other than to the other three *Lactobacillus *species, suggesting an independent adaptation to their respective niches. The independent adaptation events of *Lb. plantarum *to human gastrointestinal tract were also evidenced by transcriptome analyses [24], although *Lb. plantarum *strains isolated from the gastrointestinal tract or feces may be derived from human diet and may in fact reflect earlier adaption to other environmental niches such as fermentations of meat, plant, cheese or wine [25]. Otherwise, *Lb. brevis *is most suitable for meat fermentation in our phylogenetic tree. Given that strains of many LAB species occur in a multitude of ecological niches, further analyses of more genes and functional assays of additional LAB species are needed to reveal the molecular mechanisms underlying the adaptation of LAB species to various environmental survival niches.

**Table 1:** Summary of 28 LAB taxa and one outgroup (*Bacillus subtilis*)

Species-Organisms	Association	NCBI RefSeq
*Bacillus subtilis subsp. subtilis *str. 168	Outgroup	NC_000964
*Enterococcus faecalis *V583	gastrointestinal tract bacteria	NC_004668
*Lactobacillus acidophilus *NCFM	gastrointestinal tract bacteria	NC_006814
*Lactobacillus brevis *ATCC 367	other fermentation such as beer, wine, plants, or meat	NC_008497
*Lactobacillus casei *ATCC 334	dairy fermentation	NC_008526
*Lactobacillus casei *BL23	dairy fermentation	NC_010999
*Lactobacillus delbrueckii subsp. bulgaricus *ATCC 11842	dairy fermentation	NC_008054
*Lactobacillus delbrueckii subsp. bulgaricus *ATCC BAA-365	dairy fermentation	NC_008529
*Lactobacillus fermentum *IFO 3956	other fermentation such as beer, wine, plants, or meat	NC_010610
*Lactobacillus gasseri *ATCC 33323	gastrointestinal tract bacteria	NC_008530
*Lactobacillus helveticus *DPC 4571	dairy fermentation (Swiss cheese isolate)	NC_010080
*Lactobacillus johnsonii *NCC 533	gastrointestinal tract bacteria	NC_005362
*Lactobacillus plantarum *WCFS1	Human saliva (first), gut, dairy, wine, plants, or meat	NC_004567
*Lactobacillus reuteri *F275	gastrointestinal tract bacteria	NC_009513
*Lactobacillus reuteri *JCM 1112	gastrointestinal tract bacteria	NC_010609
*Lactobacillus sakei subsp. sakei *23K	other fermentation such as beer, wine, plants, or meat	NC_007576
*Lactobacillus salivarius *UCC118	gastrointestinal tract bacteria	NC_007929
*Lactococcus lactis subsp. cremoris *MG1363	dairy fermentation	NC_009004
*Lactococcus lactis subsp. cremoris *SK11	dairy fermentation	NC_008527
*Lactococcus lactis subsp. lactis *Il1403	dairy fermentation	NC_002662
*Leuconostoc citreum *KM20	other fermentation such as beer, wine, plants, or meat	NC_010471
*Leuconostoc mesenteroides subsp. mesenteroides *ATCC 8293	other fermentation such as beer, wine, plants, or meat	NC_008531
*Oenococcus oeni *PSU-1	other fermentation such as beer, wine, plants, or meat	NC_008528
*Pediococcus pentosaceus *ATCC 25745	dairy fermentation	NC_008525
*Streptococcus gordonii str. Challis substr*. CH1	human oral flora (dental plaque)	NC_009785
*Streptococcus mutans *UA159	oral streptococci (leading cause of dental caries)	NC_004350
*Streptococcus thermophilus *CNRZ1066	dairy fermentation	NC_006449
*Streptococcus thermophilus *LMD-9	dairy fermentation	NC_008532

### Utilities of different genes in LAB phylogeny

**Figure 2:**
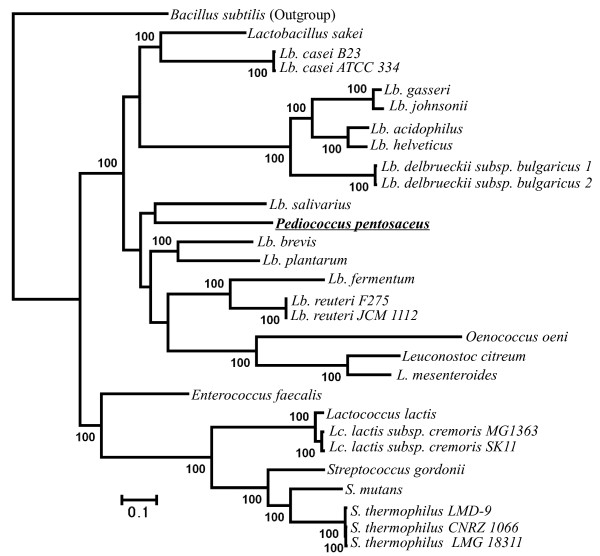
**Tree topologies inferred from 51 replication/repair/recombination genes using ML analyses with 1,000 bootstrap runs**. Bootstrap supports higher than 50 are shown above the branches. Compared to the combined tree of 232 genes (Figure 1), taxa with topological differences are underlined. *Lb. delbrueckii subsp. bulgaricus *1 = *Lb. delbrueckii subsp. bulgaricus *ATCC BAA-365; *Lb. delbrueckii subsp. bulgaricus *2 = *Lb. delbrueckii subsp. bulgaricus *ATCC 11842.

**Figure 3:**
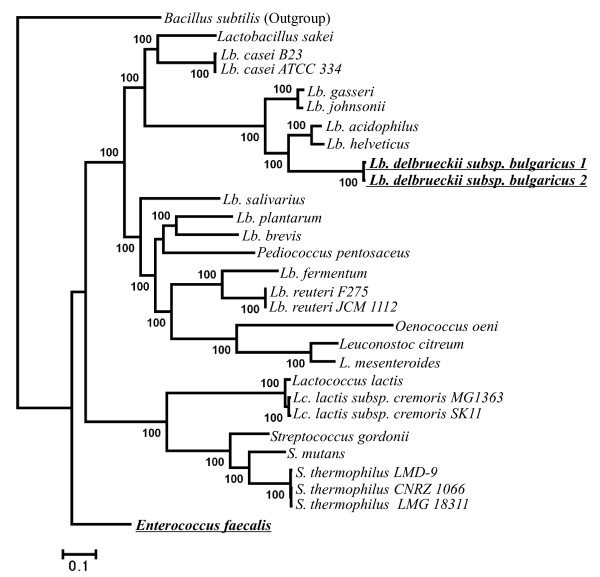
**Tree topologies inferred from 15 transcription genes using ML analyses with 1,000 bootstrap runs**. Bootstrap supports higher than 50 are shown above the branches. Compared to the combined tree of 232 genes (Figure 1), taxa with topological differences are underlined. *Lb. delbrueckii subsp. bulgaricus *1 = *Lb. delbrueckii subsp. bulgaricus *ATCC BAA-365; *Lb. delbrueckii subsp. bulgaricus *2 = *Lb. delbrueckii subsp. bulgaricus *ATCC 11842.

**Figure 4:**
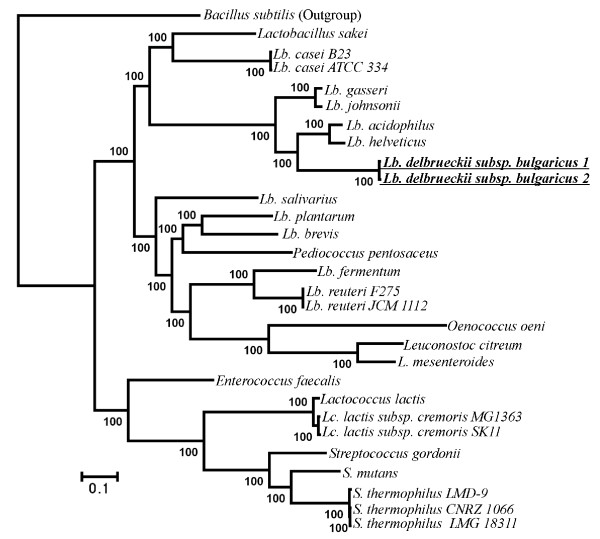
**Tree topologies inferred from 49 cellular processes and signaling genes using ML analyses with 1,000 bootstrap runs**. Bootstrap supports higher than 50 are shown above the branches. Compared to the combined tree of 232 genes (Figure 1), taxa with topological differences are underlined. *Lb. delbrueckii subsp. bulgaricus *1 = *Lb. delbrueckii subsp. bulgaricus *ATCC BAA-365; *Lb. delbrueckii subsp. bulgaricus *2 = *Lb. delbrueckii subsp. bulgaricus *ATCC 11842.

**Figure 5:**
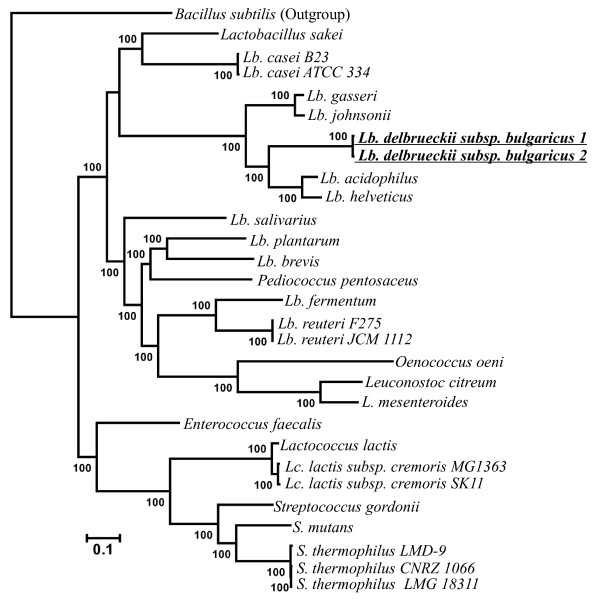
**Tree topologies inferred from 41 metabolism genes using ML analyses with 1,000 bootstrap runs**. Bootstrap supports higher than 50 are shown above the branches. Compared to the combined tree of 232 genes (Figure 1), taxa with topological differences are underlined. *Lb. delbrueckii subsp. bulgaricus *1 = *Lb. delbrueckii subsp. bulgaricus *ATCC BAA-365; *Lb. delbrueckii subsp. bulgaricus *2 = *Lb. delbrueckii subsp. bulgaricus *ATCC 11842.

**Figure 6:**
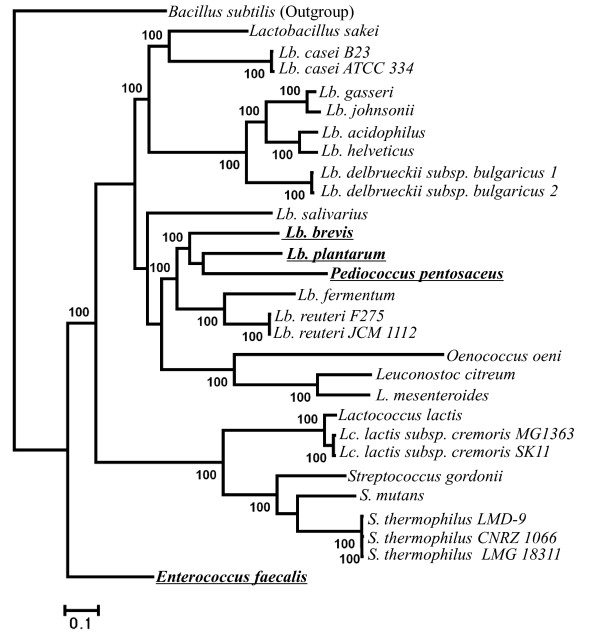
**Tree topologies inferred from 7 hypothetical genes using ML analyses with 1,000 bootstrap runs**. Bootstrap supports higher than 50 are shown above the branches. Compared to the combined tree of 232 genes (Figure 1), taxa with topological differences are underlined. *Lb. delbrueckii subsp. bulgaricus *1 = *Lb. delbrueckii subsp. bulgaricus *ATCC BAA-365; *Lb. delbrueckii subsp. bulgaricus *2 = *Lb. delbrueckii subsp. bulgaricus *ATCC 11842.

We also evaluated the phylogenetic utility of different genes used here. According to COG annotation [16], we classified 232 genes into four functional categories (Additional file 2 Table S2) relating to: information storage and processing (ISP; 135 genes), cellular processes and signaling (CPS; 49 genes), metabolism (41 genes), and hypothetical proteins (HP; 7 genes). Among them, the genes with ISP function were further divided into translation, ribosomal structure and biogenesis (TRSB; 69 genes), replication/repair/recombination (RRR; 51 genes), and transcription (15 genes). The phylogenetic analyses of LAB were repeated using each of the above six categories of genes individually. Our results suggested that the analyses of RRR (Figure 2), transcription (Figure 3), CPS (Figure 4), metabolism (Figure 5) and HP (Figure 6) genes produced different tree topologies from that of all concatenated genes (Figure 1), while the analyses of TRSB genes yielded identical tree topologies to those shown in Figure 1 suggesting that the TRSB genes are better indicators of LAB phylogeny than are other subsets of genes. The Robinson-Foulds distances analysis (Additional file 3 Table S3) also showed that there are no differences between the tree of TRSB genes and that of all concatenated genes. The differences among tree topologies based on these functional categories can be caused by various factors, including different selective constraints imposed by the functional categories that were involved in various metabolic networks [26-31].

Ranking single genes in six function categories by their respective phylogenetic resolution to LAB species reveals that 3 of 232 genes, including the ultra-violet resistance protein B gene (*uvrB*) and the DNA polymerase III gene (*polC*) from RRR category, and the penicillin binding protein 2B gene (*pbpB*) from CPS category (Additional file 2 Table S2), produced ML tree topology (Additional file 4 Figure S1a-1c) that was largely consistent with that of the complete analyses (Figure 1), albeit with low supports for some branches (BS < 70%). When we conducted the phylogenetic analyses by combining the three genes, a completely identical tree topology to that shown in Figure 1 with high supports for most of nodes was obtained. Therefore, a combined analysis using *uvrB*, *polC *and *pbpB *together seems to be a better indicator for inferring LAB phylogeny than the other subset of genes including the ribosomal protein families or RNA polymerase subunits that have been widely used in previous LAB phylogenetic studies [5-7,9,10]. The Robinson-Foulds distances analysis (Additional file 3 Table S3) also showed that there are no differences between the tree of combined *uvrB*, *polC *and *pbpB *genes and that of all concatenated genes. In the present study, the assessment of phylogenetic utility and limits of the individual genes makes it possible to preselect subsets of genes for future molecular studies of LAB phylogeny when the complete genome sequences are unavailable.

## Conclusions

In this study, phylogenetic relationships among LAB species are presented based on 232 genes from 28 LAB genome sequences. The concatenation of all these genes allowed the recovery of a strongly supported phylogeny, providing a maximum and decisive resolution of the relationships among the LAB species examined. Our phylogenomic analyses provide important insights into not only LAB phylogeny, but also the phylogenetic utility of different genes suggesting that the genes relating to translation, ribosomal structure and biogenesis (TRSB) function and a three-gene set consisting of *uvrB, polC *and *pbpB*, may be better indicators for LAB phylogenetic studies than the other subsets of genes. In addition, our study demonstrates the presence of multiple independent adaption events of LAB species to different survival habitats, indicating that further analyses of more genes from representatives of additional LAB species are needed in order to reveal the molecular mechanisms underlying the adaptation of LAB species to various environmental survival niches.

## Methods

### Sequence Data

A total of 28 available LAB genomes [6,9,32-44] representing seven genera of four families were used (Table 1). In addition, the genome sequence from *Bacillus subtilis *was used as an outgroup to root the tree.

### Identification of one-to-one orthologs for LAB phylogenetic inference

Based on protein coding genes (pseudogenes are not included) downloaded from 28 LAB and one *B. subtilis *genome sequences, a search for orthologs was conducted with the program Inparanoid version 2.0 [14]. Several stringent criteria were employed: (1) using a BLAST score cut-off of 50 bits; (2) using an overlap cut-off of 50%; (3) using a confidence value of 95% when searching in-paralogs; (4) using BLOSUM45 amino acid substitution matrix [45]. Automatic clustering of orthologs and inparalogs identified by the program Inparanoid was then performed by program Multiparanoid [15].

Among the candidate orthologous genes selected as above, we excluded those that met the following criteria from subsequent analyses: (1) lesser than 100 amino acid sequence length; (2) involved in potential horizontal gene transfer (HGT) events, as predicted by Horizontal Gene Transfer Database (HGT-DB) http://genomes.urv.es/HGT-DB/ and http://www.tinet.org/~debb/HGT/welcomeOLD.html and by previous studies [6]. In the end, a total of 232 orthologous genes, including 225 that have clear functional definition and 7 that have been annotated to be hypothetical proteins, were used to reconstruct LAB phylogeny in this study (Additional file 2 Table S2).

### Phylogenetic Reconstruction of LAB species

In total 232 orthologous genes were concatenated into two supermatrices according to two gap selection criteria in Gblocks [allowed gap positions = none (61,020 amino acids in length) and with half (only positions where 50% or more of the sequences have a gap are treated as a gap position in the final alignment) (63,910 amino acids in length)] [46]. Optimal substitution models were selected by using the program ProtTest version 2.4 [47] according to Akaike Information Criterion (AIC) [48]. The selected substitution models were used in partitioned Bayesian analysis implemented MrBayes v3.2.1 [49-51] and partitioned maximum likelihood (ML) analysis implemented in RAxML v7.0.4 [52]. The reliability of ML tree topology was evaluated by bootstrapping sampling (BP) of 1000 replicates. For Bayesian analyses, three independent runs of one-million generations each were used. The trees sampled prior to reaching convergence were discarded as burn-in and the remaining trees were used to construct the consensus tree and posterior probabilities (PP).

### Tree topology comparison

The differences between tree topologies were compared using Robinson-Foulds distances that were calculated with program Treedist from the PHYLIP v3.69 package [53].

## Authors' contributions

ZZ and PS designed the study. ZZ, ZY, LY and PS analyzed the data and wrote the manuscript. All the authors have read and approved the final manuscript.

## Supplementary Material

Additional file 1 **Table S1**. Summary of 310 one-to-one orthologs from 28 LAB species and one outgroup (*Bacillus subtilis*). Click here for file

Additional file 2 **Table S2**. Summary of 232 one-to-one orthologs used in LAB phylogenomic inference and their functional categories based on COG annotation. Click here for file

Additional file 3 **Table S3**. Robinson-Foulds distances between different tree topologies. Click here for file

Additional file 4 **Figure S1**. Single gene trees inferred from ML analyses with 1,000 replicates. Click here for file
