# Supplementary material for: Phylogenomic reconstruction of lactic acid bacteria: an update
Source: BMC Evol Biol. 2011 Jan 1;11:1. doi: 10.1186/1471-2148-11-1 (PMC3024227; doi:10.1186/1471-2148-11-1)

Figure S1. **Single gene trees inferred from ML analyses with 1,000 replicates.** Bootstrap scores for all nodes are displayed above the branches. Those taxa different from the combined tree of 232 genes (Figure 1) are bolded. *Lb. delbrueckii subsp. bulgaricus* 1 = *Lb. delbrueckii subsp. bulgaricus* ATCC BAA-365; *Lb. delbrueckii subsp. bulgaricus* 2 = *Lb. delbrueckii subsp. bulgaricus* ATCC 11842. **a,** *uvrB* gene; **b,** *polC* gene; **c,** *pbpB* gene.

Supplemental Figure 1a

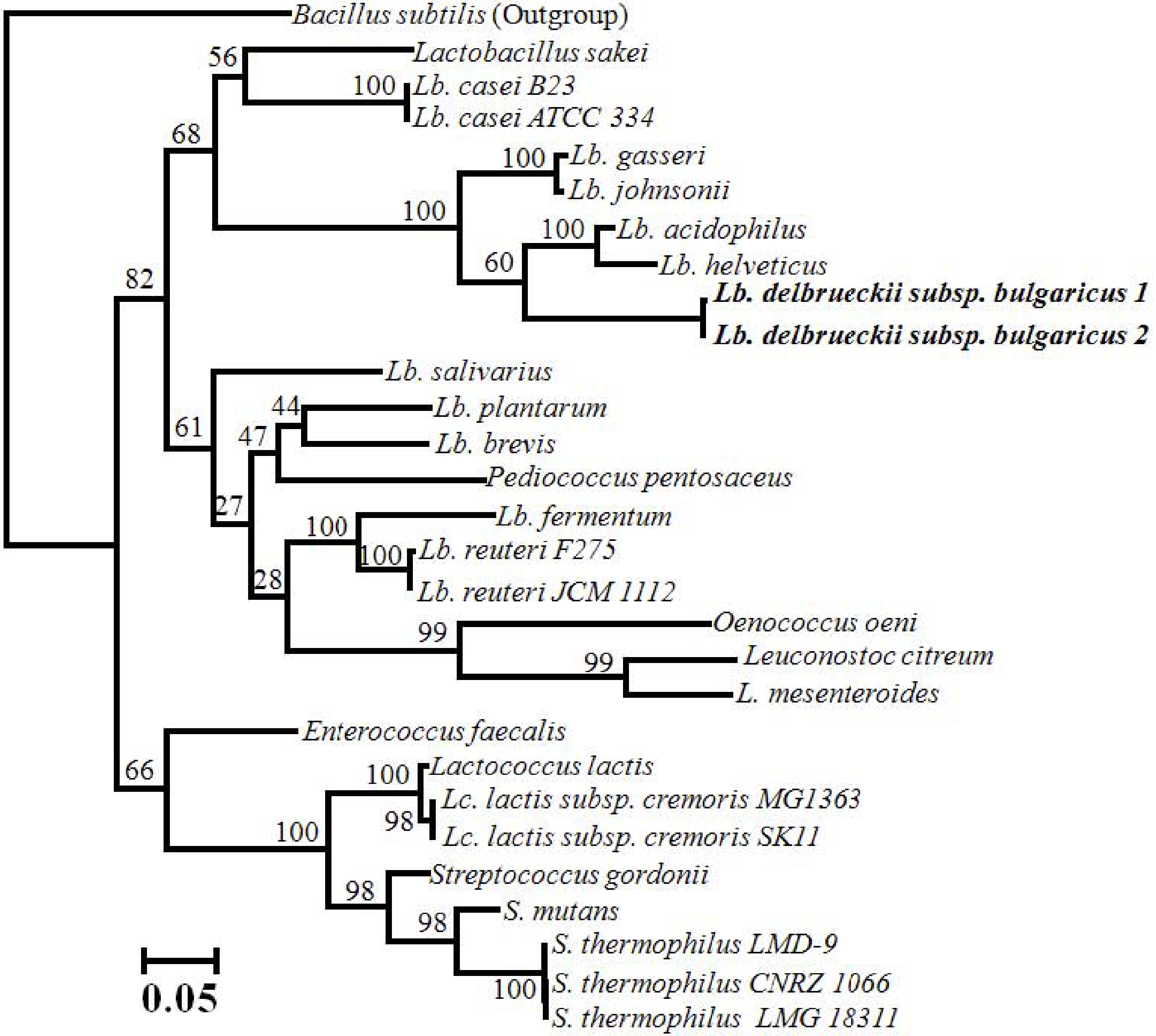

Supplemental Figure 1b

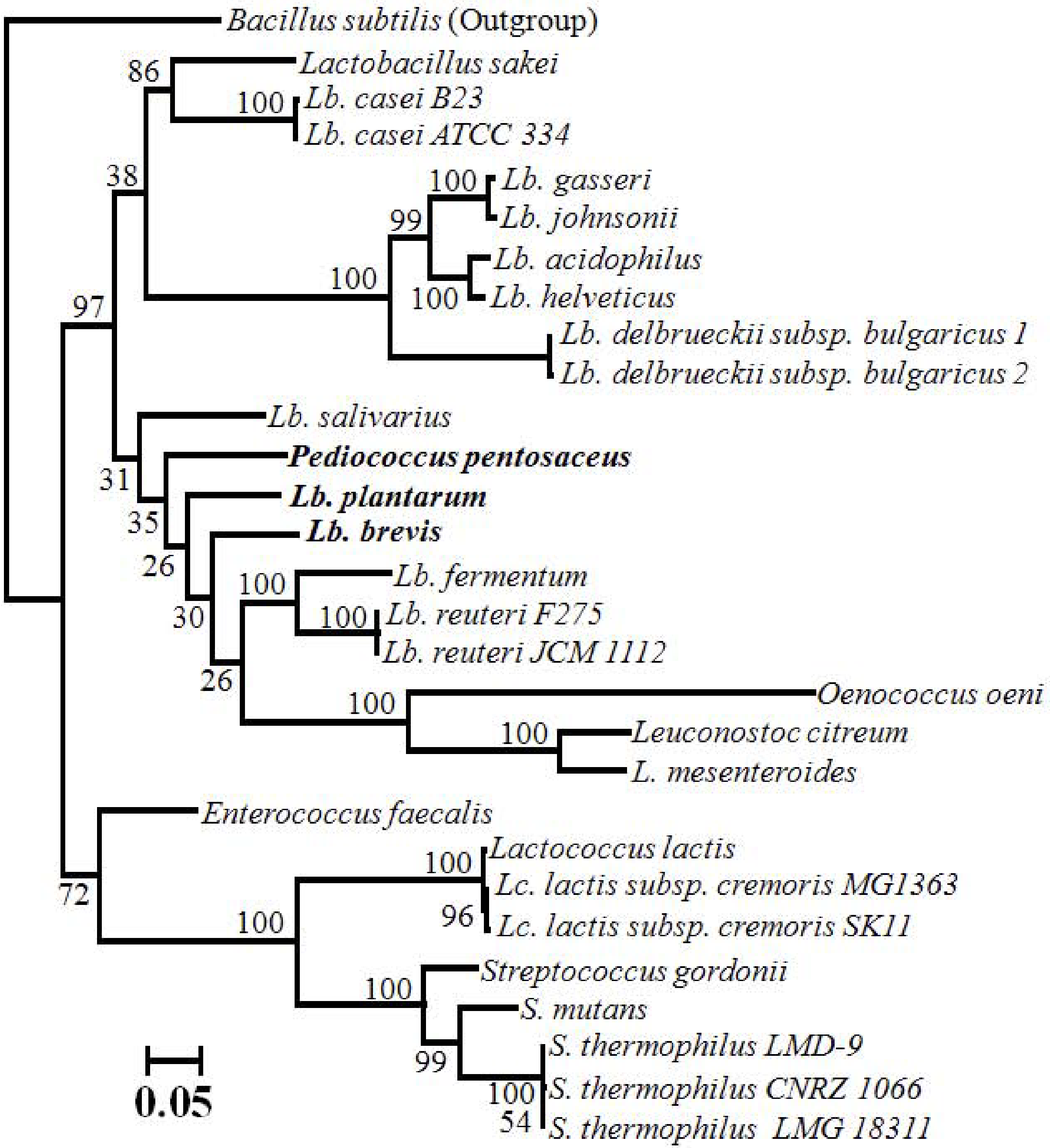

Supplemental Figure 1c

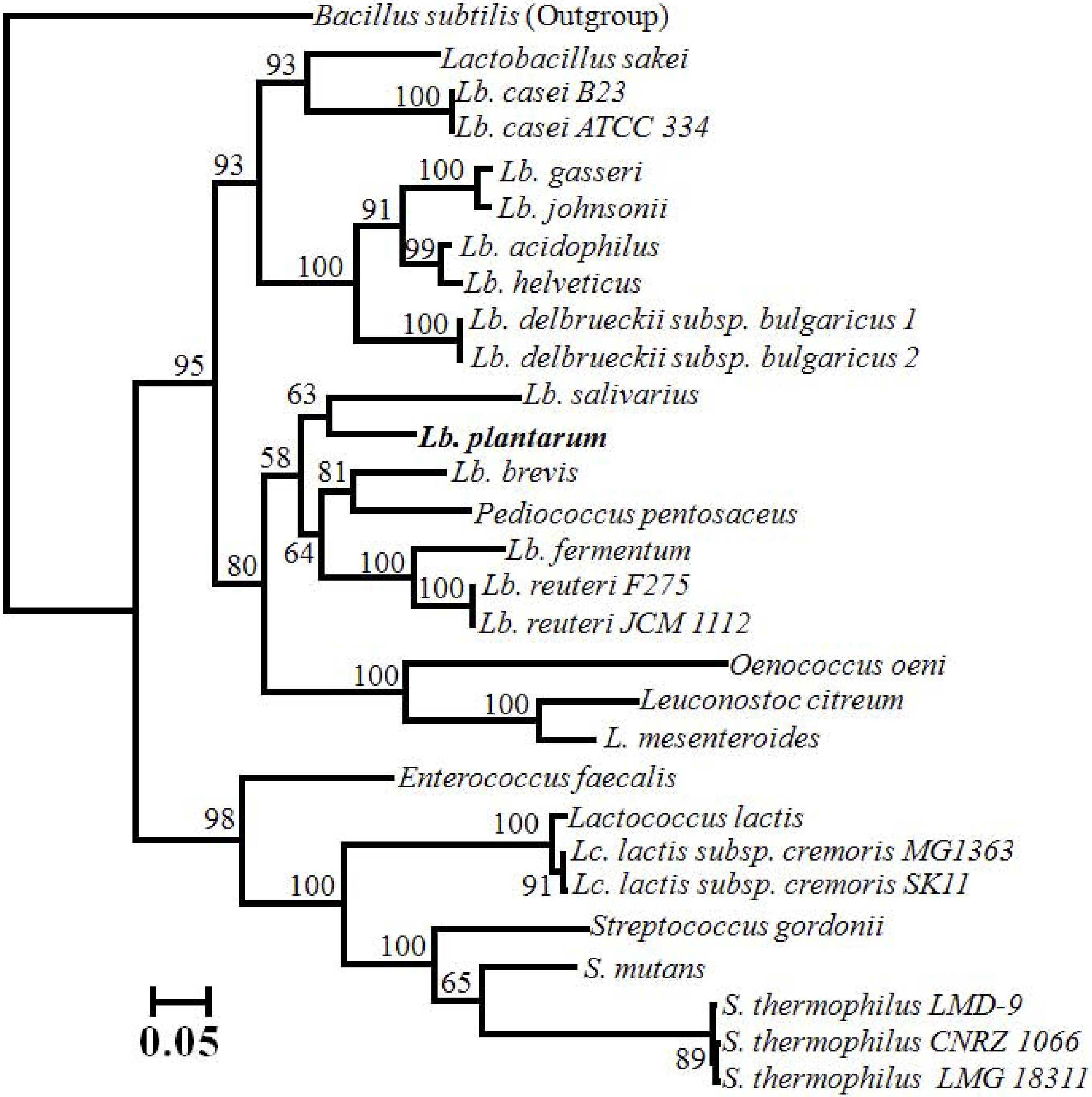

Supplement: Additional file 1 — Table S1. Summary of 310 one-to-one orthologs from 28 LAB species and one outgroup (Bacillus subtilis). [file 1471-2148-11-1-S1.PDF]
