# Supplementary material for: Phylogenomic reconstruction of lactic acid bacteria: an update
Source: BMC Evol Biol. 2011 Jan 1;11:1. doi: 10.1186/1471-2148-11-1 (PMC3024227; doi:10.1186/1471-2148-11-1)
Supplement: Additional file 3 — Table S3. Robinson-Foulds distances between different tree topologies. [file 1471-2148-11-1-S3.DOC]

**Table S3.** Robinson–Foulds distances between different tree topologies

| Tree | A | B | C | D | E | F | G | H |
| --- | --- | --- | --- | --- | --- | --- | --- | --- |
| A | 0 |  |  |  |  |  |  |  |
| B | 0 | 0 |  |  |  |  |  |  |
| C | 0 | 0 | 0 |  |  |  |  |  |
| D | 4 | 4 | 4 | 0 |  |  |  |  |
| E | 4 | 4 | 4 | 8 | 0 |  |  |  |
| F | 2 | 2 | 2 | 6 | 2 | 0 |  |  |
| G | 2 | 2 | 2 | 6 | 2 | 0 | 0 |  |
| H | 6 | 6 | 6 | 10 | 6 | 8 | 8 | 0 |

Robinson–Foulds distances represent the number of bipartitions that are present in one tree and absent in the other. A, 232 concatenated genes; B, concatenated three genes, *uvrB*, *polC* and *pbpB*; C, TRSB; D, RRR; E, Transcription; F, CPS; G, Metabolism; H, HP.
